# Supplementary material for: Unraveling the evolutionary origin of the complex Nuclear Receptor Element (cNRE), a cis-regulatory module required for preferential expression in the atrial chamber
Source: Commun Biol. 2024 Apr 4;7:371. doi: 10.1038/s42003-024-05972-6 (PMC10995137; doi:10.1038/s42003-024-05972-6)
Supplement: Supplementary file 2 — Supplementary information [file 42003_2024_5972_MOESM2_ESM.pdf]

Unraveling the evolutionary origin of the complex Nuclear Receptor Element (cNRE), a *cis*-regulatory module required for preferential expression in the atrial chamber

Luana Nunes Santos[1,2,3], Ângela Maria Sousa Costa[1], Martin Nikolov[2], João E. Carvalho[4], Allysson Coelho Sampaio[3,5], Frank E. Stockdale[6], Gang Feng Wang[ø], Hozana Andrade Castillo[1,2], Mariana Bortoletto Grizante[1], Stefanie Dudczig[7], Michelle Vasconcelos[3], Nadia Rosenthal[8,9], Patricia Regina Jusuf[7], Hieu T. Nim[10], Paulo de Oliveira[1], Tatiana Guimarães de Freitas Matos[3], William Nikovits Jr.[6], Izabella Luisa Tambones[1], Ana Carolina Migliorini Figueira[1], Michael Schubert[4], Mirana Ramialison[2,10,*], and José Xavier-Neto[11,12,*]

Supplementary material

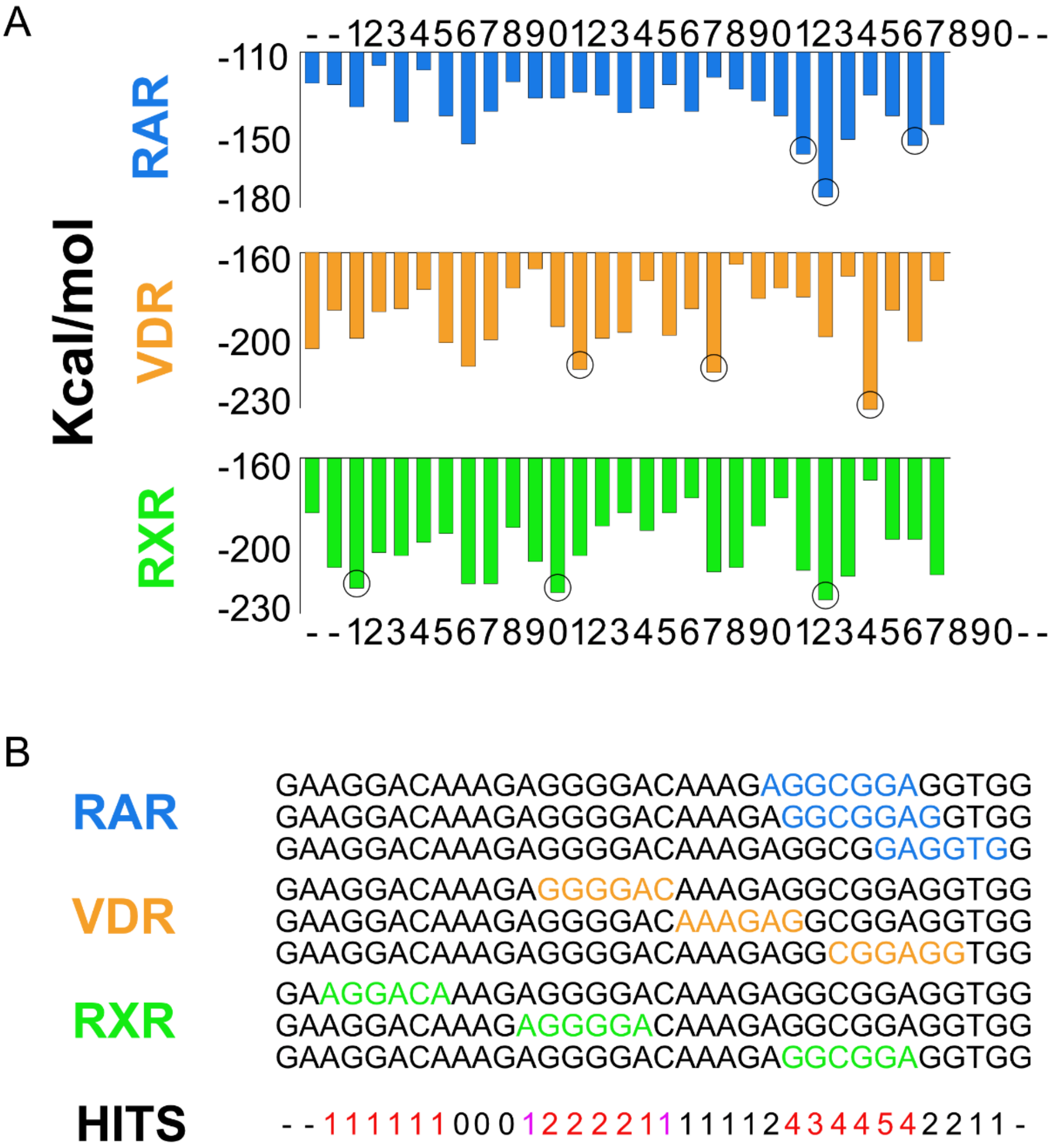

Supplementary Figure S1. Computerized profiling of nuclear receptor binding sites (hexads) at the complex Nuclear Receptor Element (cNRE). A) The cNRE DNA was scanned with nuclear receptor monomers and interaction energies were plotted for each

hexad as surrogates for affinities. Energies are represented as bars aligned to the first nucleotide of a hexad. The energies below the 10th percentile are marked by empty circles and nucleotides corresponding to these values are displayed in blue, orange, and green for RAR, VDR, and RXR, respectively. **B)** The frequency a given cNRE nucleotide was included in affinity hexads was scored (hits) to assess nuclear receptor binding potential within the cNRE.

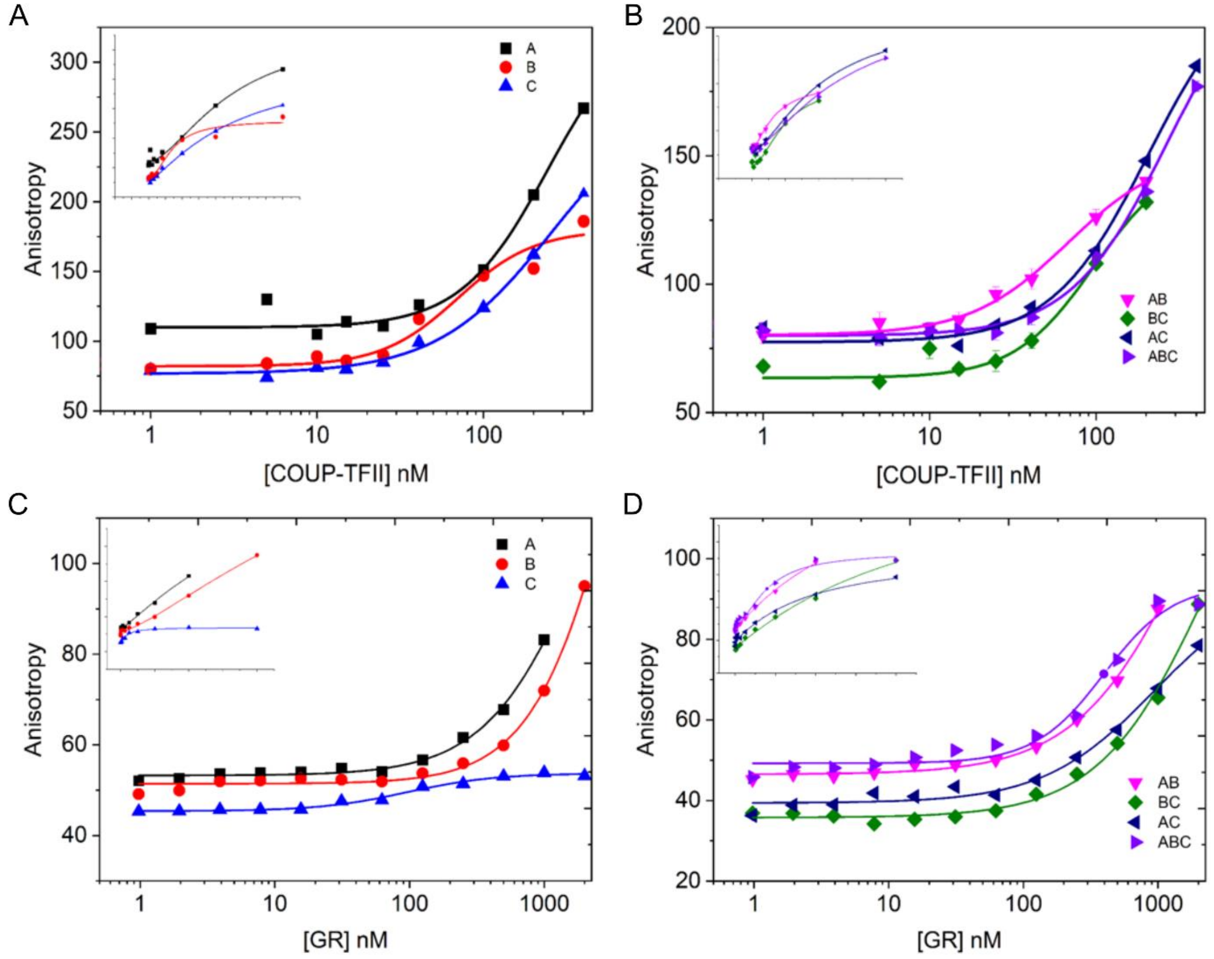

Supplementary Figure S2. Selective binding of nuclear receptors to the complex nuclear receptor element (cNRE). Affinity curves for the nuclear receptors COUPTF-II (COUP transcription factor 2) and GR (glucocorticoid receptor) with Hexads A, B, and C

of the cNRE. **A)** Normalized fluorescence anisotropy curves established for COUPTF-II and Hexads A, B, and C. **B)** Normalized fluorescence anisotropy curves established for COUPTF-II and Hexad combinations (AB, BC, AC, and ABC). **C)** Normalized fluorescence anisotropy curves established for GR and Hexads A, B, and C. **D)** Normalized fluorescence anisotropy curves established for GR and Hexad combinations (AB, BC, AC, and ABC). Insets show graphs with a non-logarithmic scale for the assayed nuclear receptor concentrations.

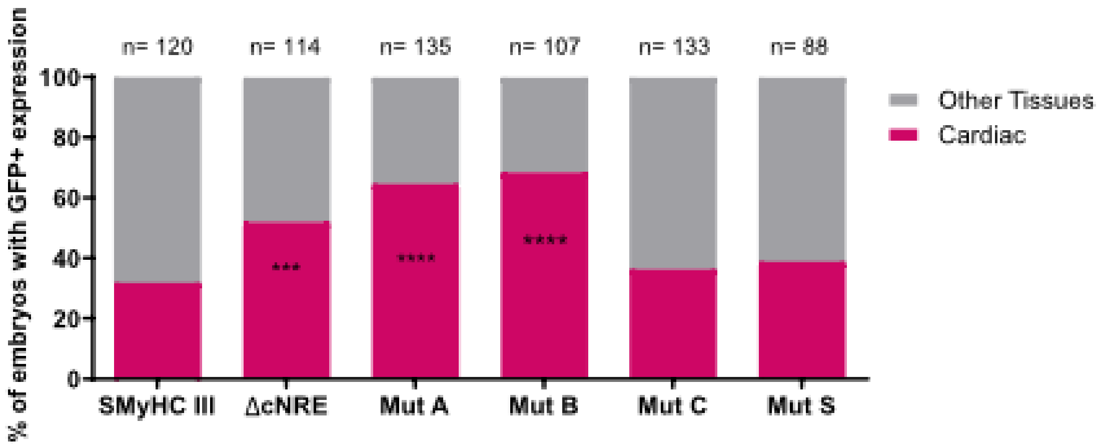

Supplementary Figure S3. eGFP expression in cardiac and non-cardiac tissues of zebrafish embryos following injection with different constructs of the *SMyHC III* promoter. Graphic representation of eGFP expression in cardiac and other tissues of cohorts of zebrafish embryos injected with different *SMyHC III* promoter constructs and analyzed at 48 hours post fertilization (hpf). chi-square test, *** 0.0009, **** $p<0.0001$, comparing *SMyHC III* to each mutation and condition.

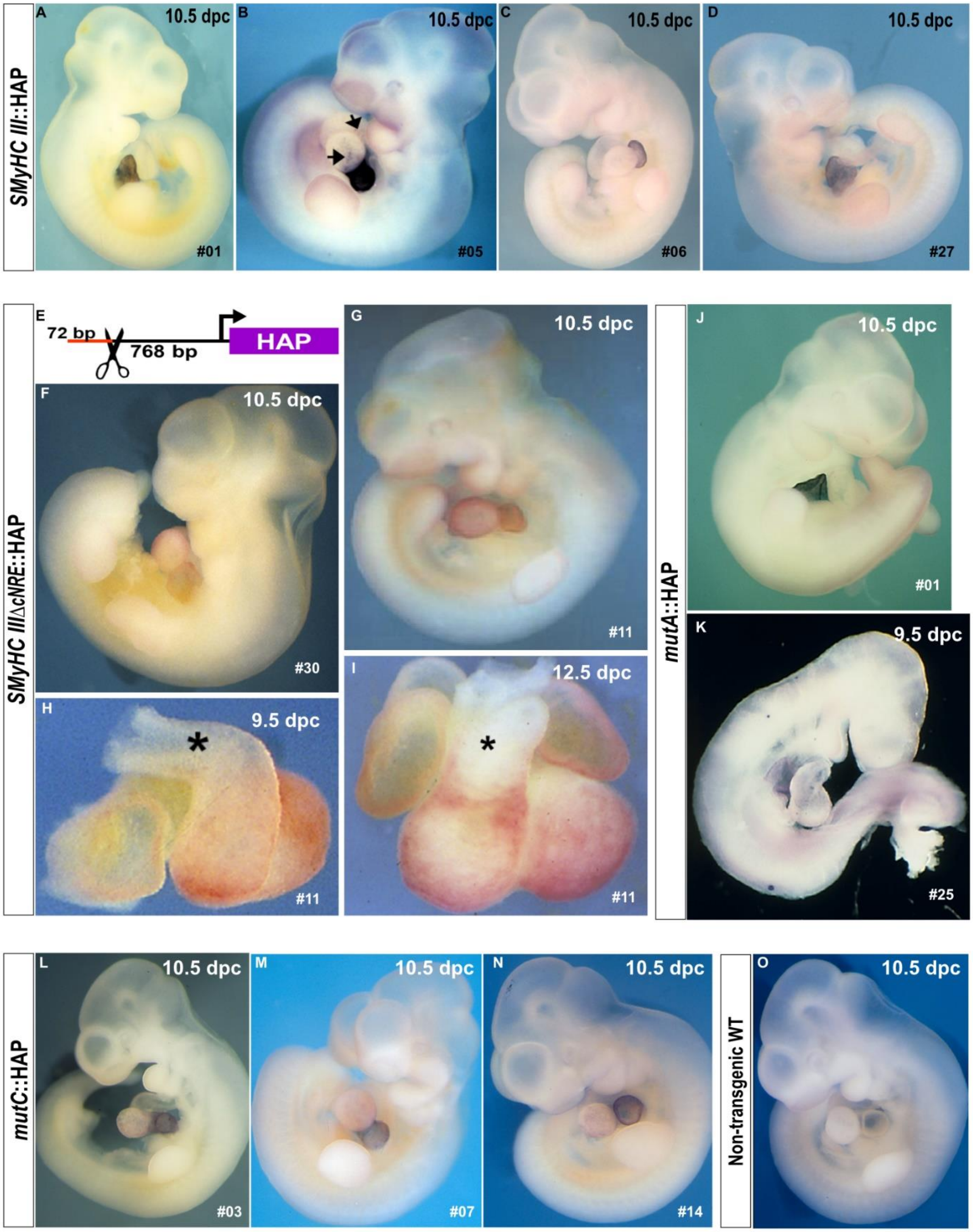

SMyHC III::HAP
A
10.5 dpc
#01
B
10.5 dpc
#05
C
10.5 dpc
#06
D
10.5 dpc
#27
SMyHC IIIΔcNRE::HAP
E
72 bp
768 bp
HAP
F
10.5 dpc
#30
G
10.5 dpc
#11
H
9.5 dpc
*
#11
I
12.5 dpc
*
#11
mutA::HAP
J
10.5 dpc
#01
K
9.5 dpc
#25
mutC::HAP
L
10.5 dpc
#03
M
10.5 dpc
#07
N
10.5 dpc
#14
Non-transgenic WT
O
10.5 dpc

Supplementary Figure S4. Expression of HAP in *SMyHC III*::HAP embryos and mutants. A-D) HAP expression in *SMyHC III*::HAP at 10.5 days post coitum (dpc) in mouse lines 1, 5, 6, and 27, respectively. Arrows indicate the ventricle. **E)** Strategy for the deletion of the distal 72 bp of the *SMyHC III* promoter. **F,G)** HAP expression in *SMyHC IIIΔcNRE*::HAP at 10.5 dpc in mouse lines 30 and 11, respectively. **H,I)** HAP expression in isolated embryonic hearts of *SMyHC IIIΔcNRE*::HAP mouse line 11 at 9.5 dpc. and at 12.5 dpc, respectively. "*" marks the distal outflow tract. **J)** HAP expression in *mutA*::HAP (mutation of Hexad A) at 10.5 dpc in mouse line 1 . **K)** HAP expression in *mutA:*:HAP (mutation of Hexad A) at 9.5 dpc in mouse line 25. **L-N)** HAP expression in *mutC*::HAP (mutation of Hexad C) at 10.5 dpc in mouse lines 3, 7, and 14, respectively. **O)** A wild-type (WT) non-transgenic embryo displaying background HAP staining at 10.5 dpc.

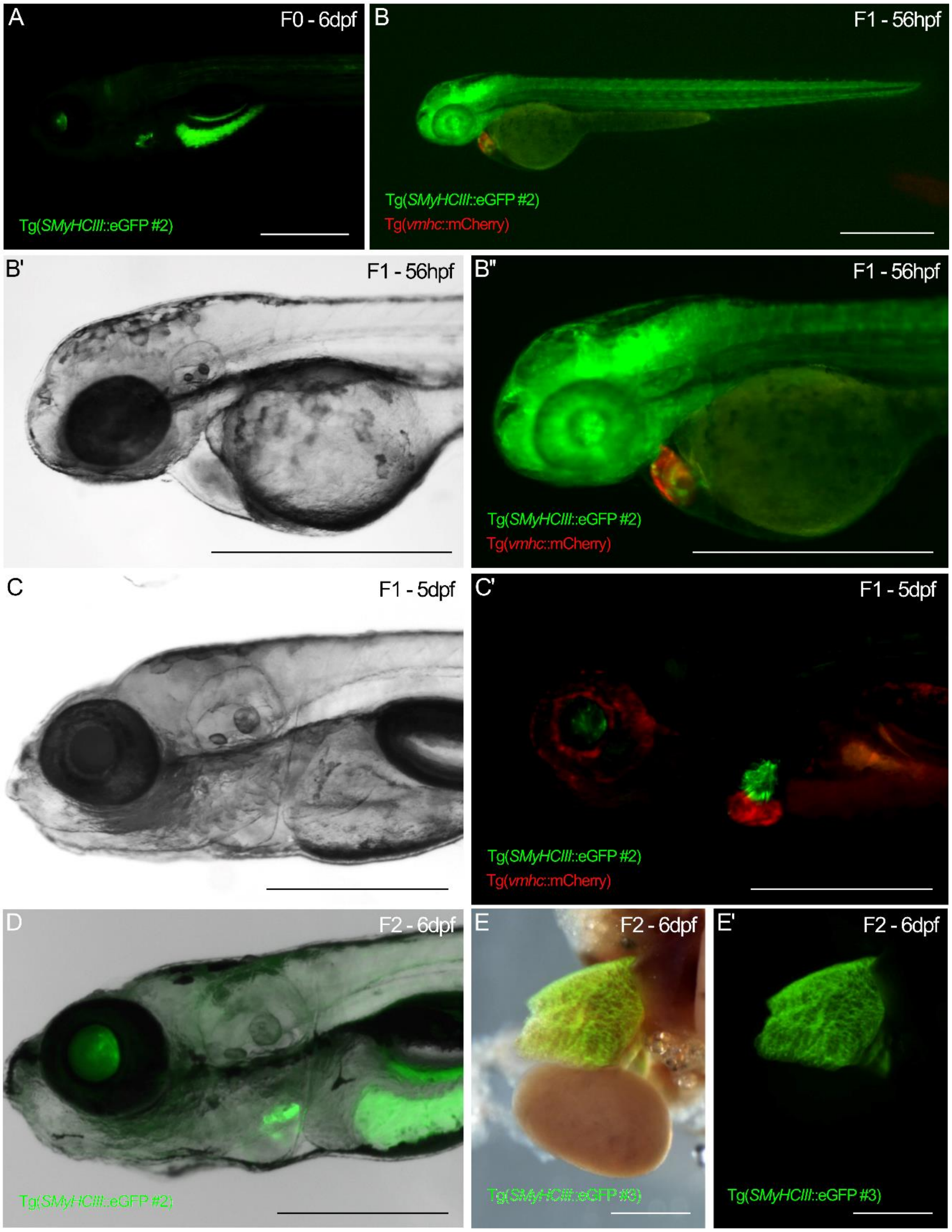

Supplementary Figure S5. The *SMyHC III* promoter induces stable preferential atrial expression in zebrafish. A) Stable transgenic *SMyHC III*::eGFP (green) female F0

founder at 6 days post fertilization (dpf). **B)** Stable transgenic *SMyHC III*::eGFP (green) F1 on a *vmhc*::mCherry (red) background at 56 hours post fertilization (hpf), showing the specimen under fluorescent light with an overlay of the green and red channels. **B')** Brightfield image of the anterior region of the animal shown in (B). **B'')** Fluorescent image of the anterior region of the animal shown in (B) showing an overlay of the green and red channels. **C)** Brightfield image of the anterior region of a stable transgenic *SMyHC III*::eGFP F1 on a *vmhc*::mCherry background at 5dpf. **C')** Fluorescent image of the animal shown in (C) showing an overlay of the green and red channels, with the *SMyHC III*::eGFP signal in green and the *vmhc*::mCherry signal in red. **D)** Brightfield/fluorescence overlay of the anterior region of a stable transgenic *SMyHC III*::eGFP (green) F2 at 6 dpf. **E)** Isolated heart from a stable transgenic *SMyHC III*::eGFP (green) F2 line at 6 dpf embryo with brightfield/fluorescence overlay. **E')** same specimen as in (E) showing green fluorescence only. Scale bars are 500 µm in (A-D) and 100 µm in (E,E').

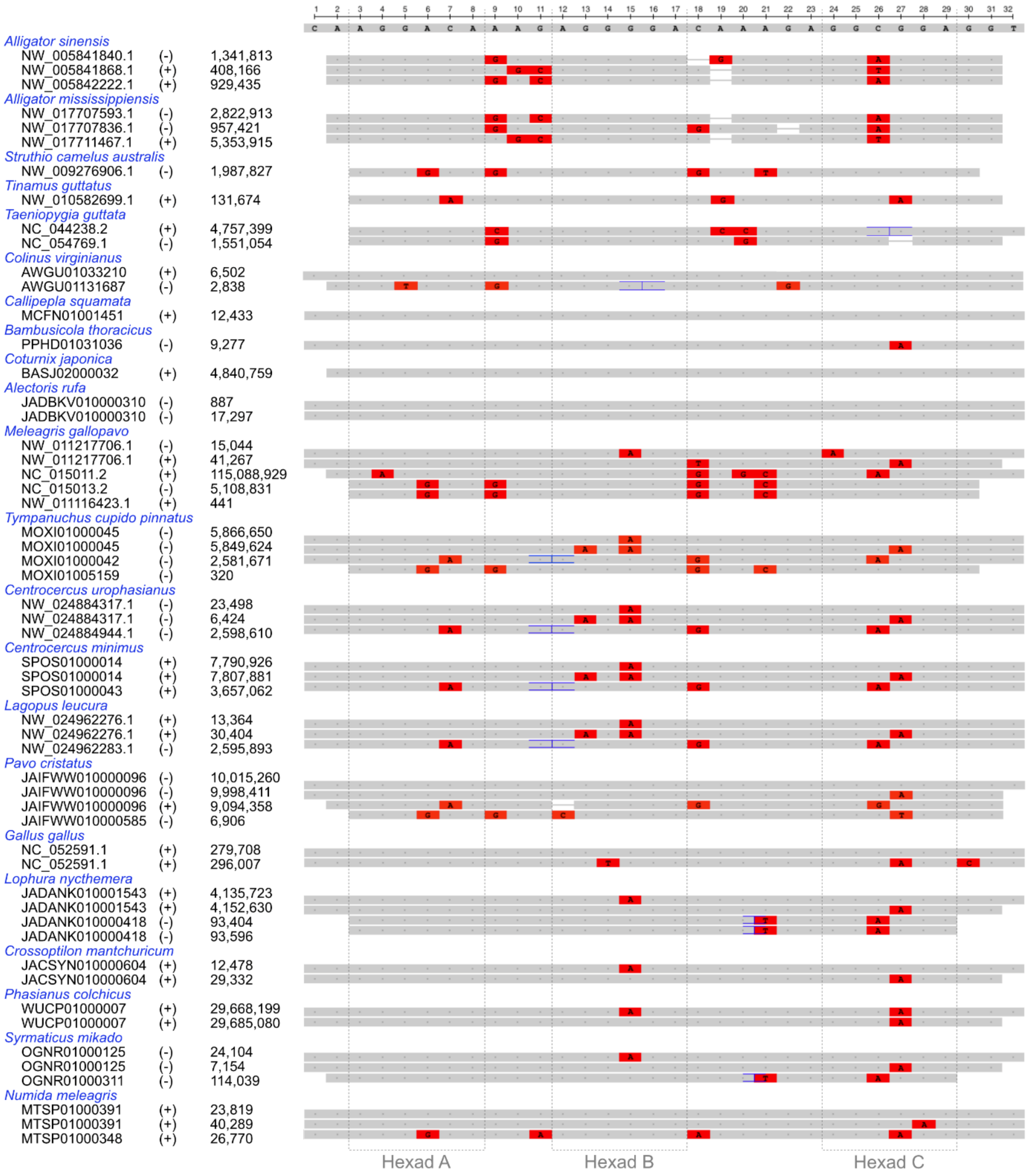
Alligator sinensis
NW_005841840.1 (-) 1,341,813
NW_005841868.1 (+) 408,166
NW_005842222.1 (+) 929,435
Alligator mississippiensis
NW_017707593.1 (-) 2,822,913
NW_017707836.1 (-) 957,421
NW_017711467.1 (+) 5,353,915
Struthio camelus australis
NW_009276906.1 (-) 1,987,827
Tinamus guttatus
NW_010582699.1 (+) 131,674
Taeniopygia guttata
NC_044238.2 (+) 4,757,399
NC_054769.1 (-) 1,551,054
Colinus virginianus
AWGU01033210 (+) 6,502
AWGU01131687 (-) 2,838
Callipepla squamata
MCFN01001451 (+) 12,433
Bambusicola thoracicus
PPHD01031036 (-) 9,277
Coturnix japonica
BASJ02000032 (+) 4,840,759
Alectoris rufa
JADBKV010000310 (-) 887
JADBKV010000310 (-) 17,297
Meleagris gallopavo
NW_011217706.1 (-) 15,044
NW_011217706.1 (+) 41,267
NC_015011.2 (+) 115,088,929
NC_015013.2 (-) 5,108,831
NW_011116423.1 (+) 441
Tympanuchus cupido pinnatus
MOXI01000045 (-) 5,866,650
MOXI01000045 (-) 5,849,624
MOXI01000042 (-) 2,581,671
MOXI01005159 (-) 320
Centrocercus urophasianus
NW_024884317.1 (-) 23,498
NW_024884317.1 (-) 6,424
NW_024884944.1 (-) 2,598,610
Centrocercus minimus
SPOS01000014 (+) 7,790,926
SPOS01000014 (+) 7,807,881
SPOS01000043 (+) 3,657,062
Lagopus leucura
NW_024962276.1 (+) 13,364
NW_024962276.1 (+) 30,404
NW_024962283.1 (-) 2,595,893
Pavo cristatus
JAIFWW010000096 (-) 10,015,260
JAIFWW010000096 (-) 9,998,411
JAIFWW010000096 (+) 9,094,358
JAIFWW010000585 (-) 6,906
Gallus gallus
NC_052591.1 (+) 279,708
NC_052591.1 (+) 296,007
Lophura nycthemera
JADANK010001543 (+) 4,135,723
JADANK010001543 (+) 4,152,630
JADANK010000418 (-) 93,404
JADANK010000418 (-) 93,596
Crossoptilon mantchuricum
JACSYN010000604 (+) 12,478
JACSYN010000604 (+) 29,332
Phasianus colchicus
WUCP01000007 (+) 29,668,199
WUCP01000007 (+) 29,685,080
Syrmaticus mikado
OGNR01000125 (-) 24,104
OGNR01000125 (-) 7,154
OGNR01000311 (-) 114,039
Numida meleagris
MTSP01000391 (+) 23,819
MTSP01000391 (+) 40,289
MTSP01000348 (+) 26,770
Hexad A
Hexad B
Hexad C

Supplementary Figure S6. cNRE and cNRE-like sequences identified in selected Archosauria genomes. For each cNRE-like hit, the accession number of the chromosome/scaffold is indicated as well as the orientation and the position of the cNRE-like sequence. In addition, a representation of the cNRE-like sequence is provided, with mismatches marked in red, deletions with a thin grey line, and insertions with blue brackets (each relative to the *Coturnix coturnix* 5’ cNRE sequence).

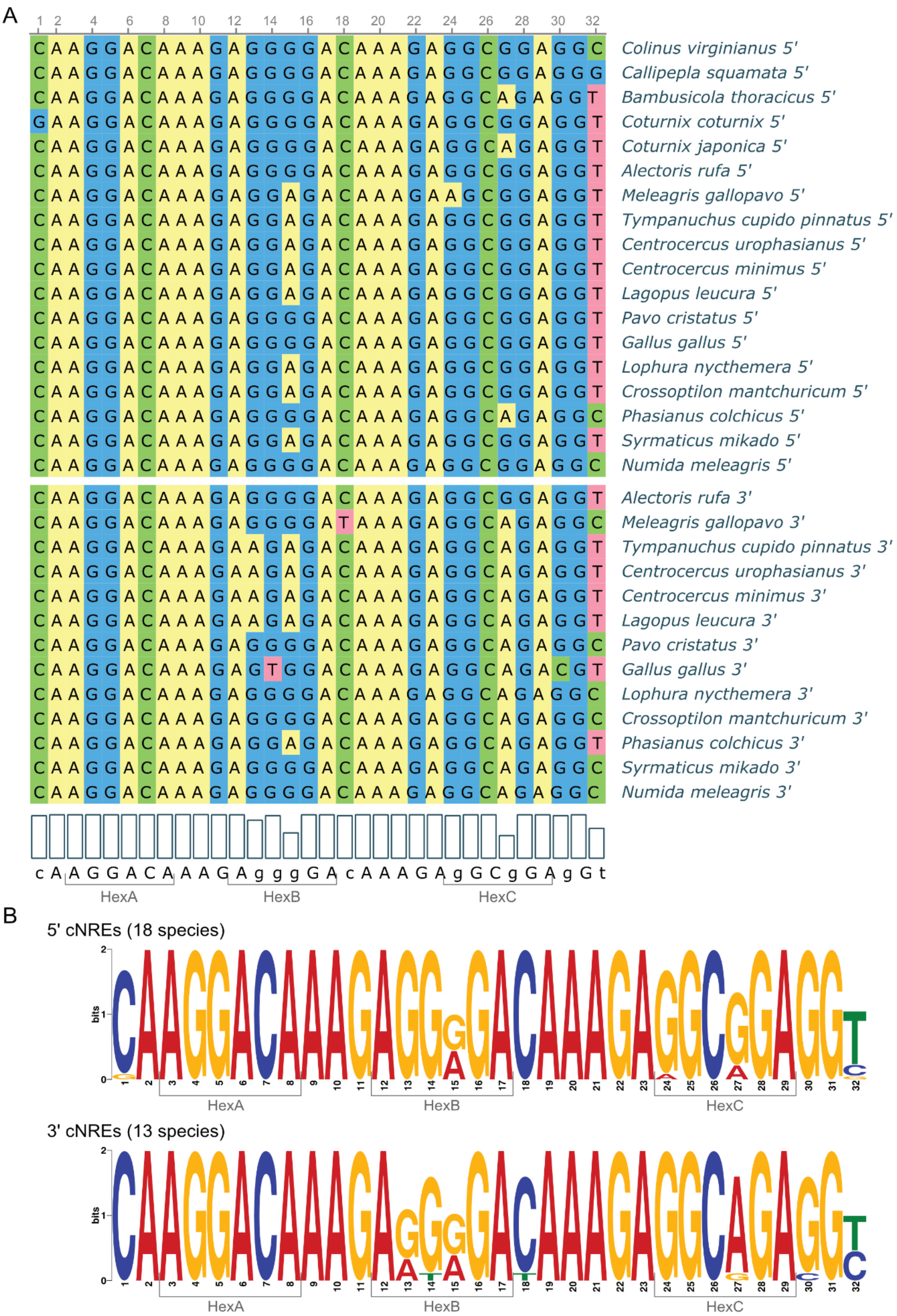
A
Colinus virginianus 5'
Callipepla squamata 5'
Bambusicola thoracicus 5'
Coturnix coturnix 5'
Coturnix japonica 5'
Alectoris rufa 5'
Meleagris gallopavo 5'
Tympanuchus cupido pinnatus 5'
Centrocercus urophasianus 5'
Centrocercus minimus 5'
Lagopus leucura 5'
Pavo cristatus 5'
Gallus gallus 5'
Lophura nycthemera 5'
Crossoptilon mantchuricum 5'
Phasianus colchicus 5'
Syrmaticus mikado 5'
Numida meleagris 5'
Alectoris rufa 3'
Meleagris gallopavo 3'
Tympanuchus cupido pinnatus 3'
Centrocercus urophasianus 3'
Centrocercus minimus 3'
Lagopus leucura 3'
Pavo cristatus 3'
Gallus gallus 3'
Lophura nycthemera 3'
Crossoptilon mantchuricum 3'
Phasianus colchicus 3'
Syrmaticus mikado 3'
Numida meleagris 3'
c A A G G A C A A A G A g g g G A c A A A G A g G C g G A g G t
HexA
HexB
HexC
B
5' cNREs (18 species)
bits
3' cNREs (13 species)

Supplementary Figure S7. The cNRE sequences of Galliformes. A) Alignment of 5' (top) and 3' (bottom) cNREs in different galliform bird species. **B)** Consensus sequences for the 5' and the 3' cNREs.

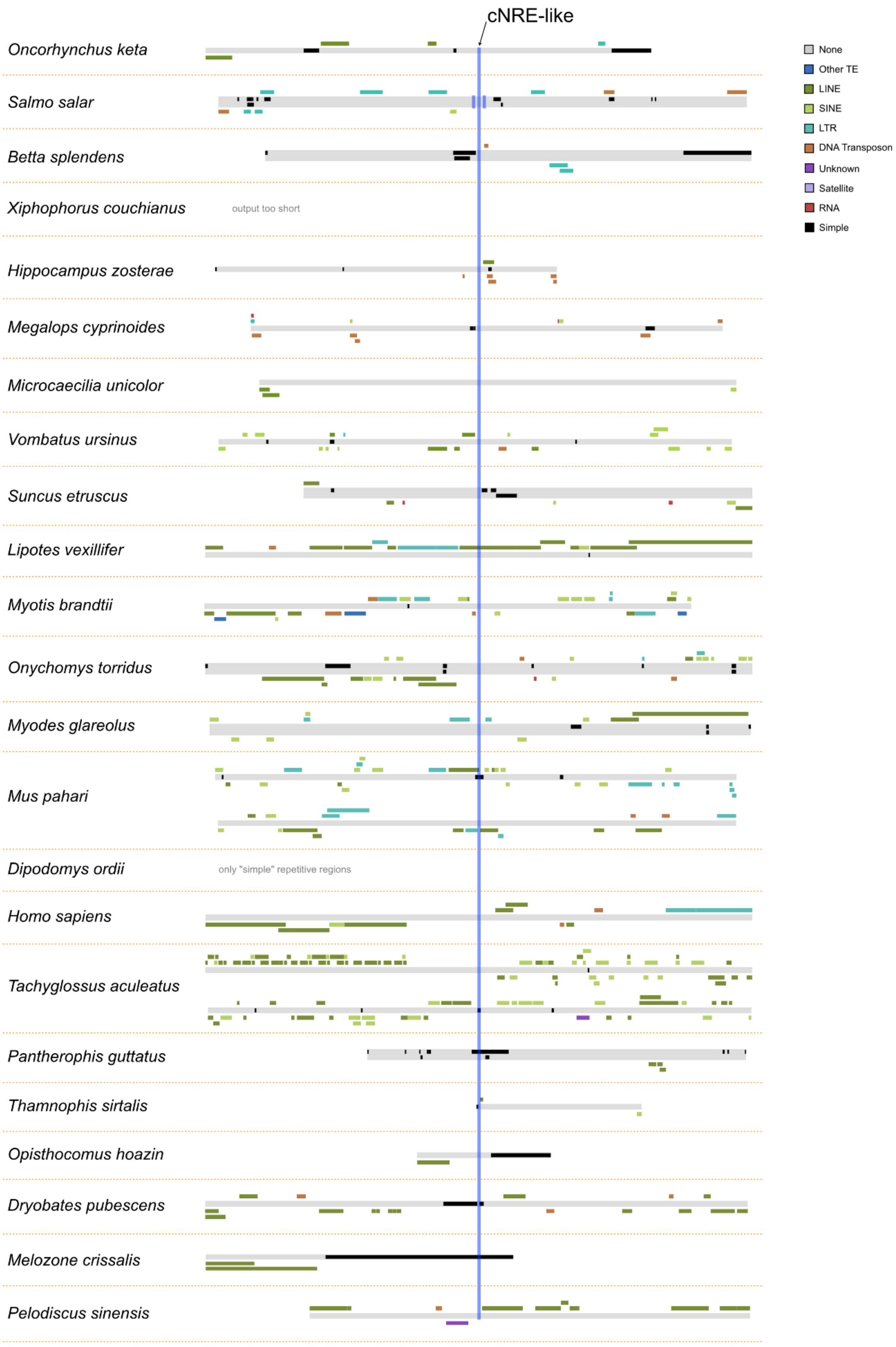

cNRE-like
Oncorhynchus keta
Salmo salar
Betta splendens
Xiphophorus couchianus
output too short
Hippocampus zosterae
Megalops cyprinoides
Microcaecilia unicolor
Vombatus ursinus
Suncus etruscus
Lipotes vexillifer
Myotis brandtii
Onychomys torridus
Myodes glareolus
Mus pahari
Dipodomys ordii
only "simple" repetitive regions
Homo sapiens
Tachyglossus aculeatus
Pantherophis guttatus
Thamnophis sirtalis
Opisthocomus hoazin
Dryobates pubescens
Melozone crissalis
Pelodiscus sinensis
None
Other TE
LINE
SINE
LTR
DNA Transposon
Unknown
Satellite
RNA
Simple

Supplementary Figure S8. Transposable elements and repetitive sequences in the vicinity of cNRE-like sequences in different vertebrate genomes (excluding those of Galliformes). Graphical representation of the Dfam results analyzing regions upstream and downstream of cNRE-like hits.

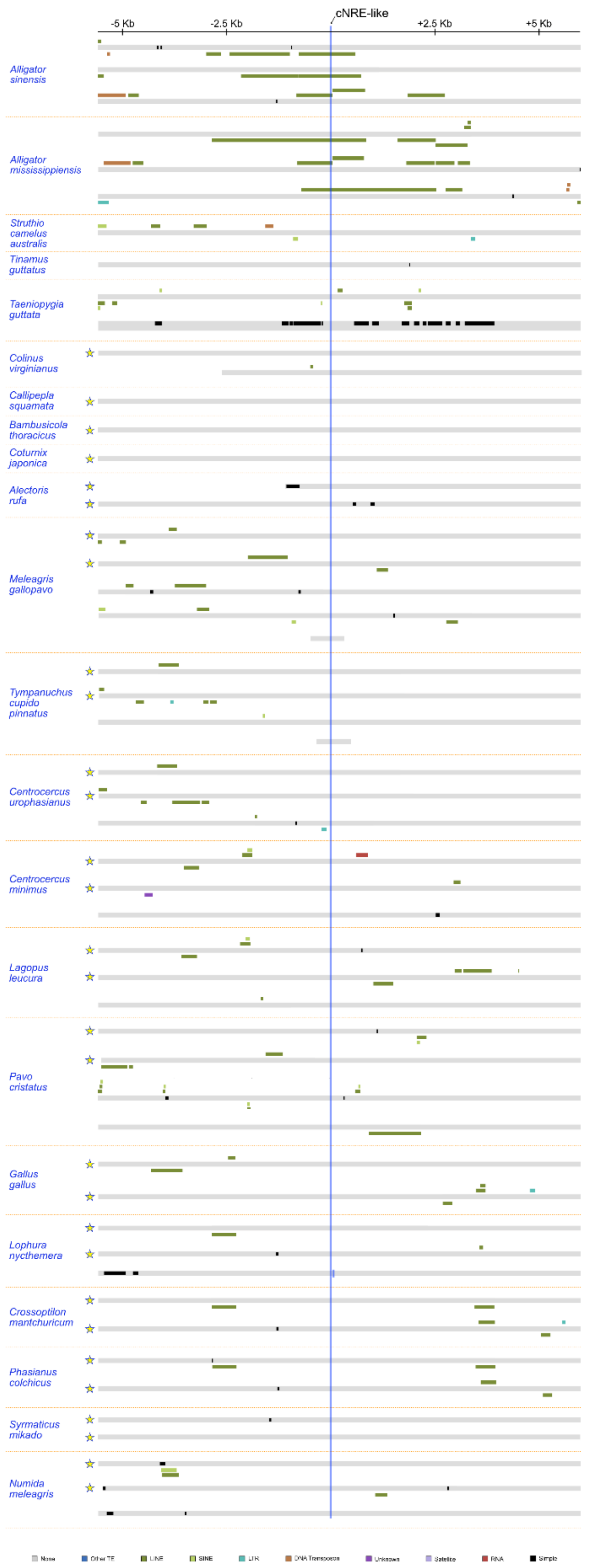

cNRE-like
-5 Kb
-2.5 Kb
+2.5 Kb
+5 Kb
Alligator sinensis
Alligator mississippiensis
Struthio camelus australis
Tinamus guttatus
Taeniopygia guttata
Colinus virginianus
Callipepla squamata
Bambusicola thoracicus
Coturnix japonica
Alectoris rufa
Meleagris gallopavo
Tympanuchus cupido pinnatus
Centrocercus urophasianus
Centrocercus minimus
Lagopus leucura
Pavo cristatus
Gallus gallus
Lophura nycthemera
Crossoptilon mantchuricum
Phasianus colchicus
Syrmaticus mikado
Numida meleagris
None
Other TE
LINE
SINE
LTR
DNA Transposon
Unknown
Satellite
RNA
Simple

Supplementary Figure S9. Transposable elements and repetitive sequences in the vicinity of cNRE-like sequences in selected Archosauria genomes. Graphical representation of the Dfam results analyzing regions upstream and downstream of cNRE-like hits. Yellow stars indicate cNRE sequences with less than 4 mismatches relative to the *Coturnix coturnix* 5' cNRE sequence.

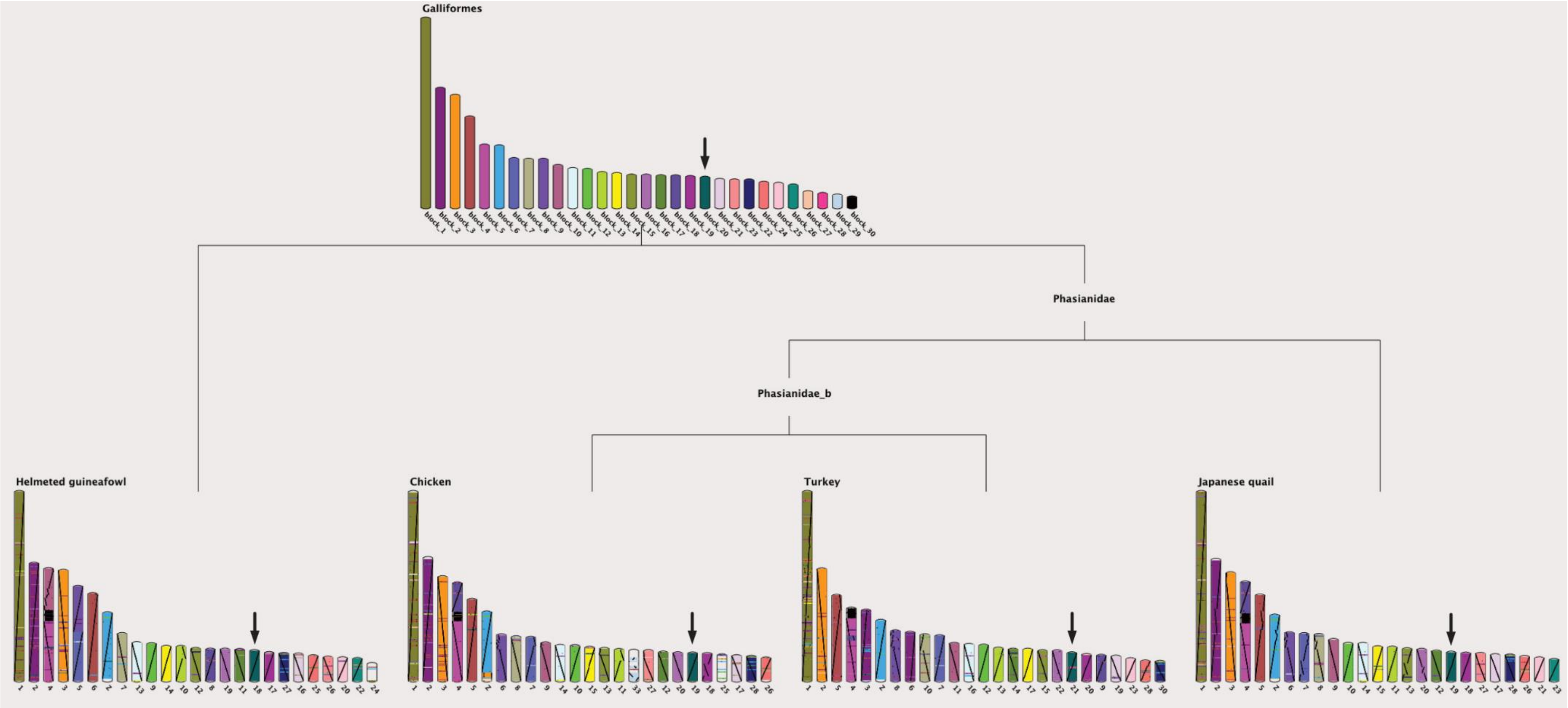
Galliformes
Phasianidae
Phasianidae_b
Helmeted guineafowl
Chicken
Turkey
Japanese quail

Supplementary Figure S10. Chromosome evolution in Galliformes based on gene synteny. Colors indicate regions homologous to predicted syntenic blocks of the genome of the last common ancestor of galliform birds. Arrows indicate homologous genome regions containing the cNREs and the *SMyHC III* gene in the galliform ancestor, the helmeted guineafowl (*Numida meleagris*), the chicken (*Gallus gallus*), the wild turkey (*Meleagris gallopavo*), and the Japanese quail (*Coturnix japonica*).

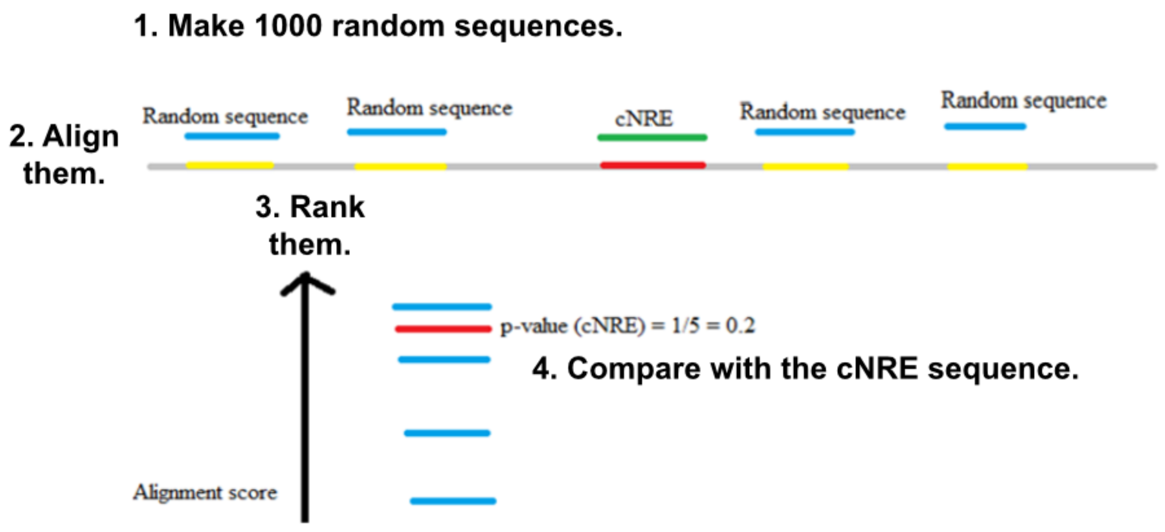

Supplementary Figure S11. The R-based pairwise alignment method for calculating p-values for viral hits. 1,000 random sequences were produced and their pairwise alignment scores compared to those obtained with the cNRE. A viral hit was considered as statistically significant, when the pairwise alignment score of the cNRE sequence was more than 95% higher than the score obtained with random sequences.

Supplementary Data 1: Raw data of HAP activity in cardiac tissues (Figures 1 and 2).

Supplementary Data 2: Accession numbers for amino acid sequences used in phylogenetic analyses.

Supplementary Data 3: BLAST results for cNRE-like hits in vertebrate genomes (excluding those of Galliformes).

Supplementary Data 4: Final MYH6, MYH7, and MYH7B amino acid alignment used to calculate phylogenetic trees.
