# Supplementary material for: Unraveling the evolutionary origin of the complex Nuclear Receptor Element (cNRE), a cis-regulatory module required for preferential expression in the atrial chamber
Source: Commun Biol. 2024 Apr 4;7:371. doi: 10.1038/s42003-024-05972-6 (PMC10995137; doi:10.1038/s42003-024-05972-6)
Supplement: Supplementary file 3 — Description of Additional Supplementary Files [file 42003_2024_5972_MOESM3_ESM.pdf]

Description of Additional Supplementary Files

File name: Supplementary Data 1
Description: Raw data of HAP activity in cardiac tissues (Figures 1 and 2).

File name: Supplementary Data 2
Description: Accession numbers for amino acid sequences used in phylogenetic analyses.

File name: Supplementary Data 3
Description: BLAST results for cNRE-like hits in vertebrate genomes (excluding those of Galliformes).

File name: Supplementary Data 4
Description: Final MYH6, MYH7, and MYH7B amino acid alignment used to calculate phylogenetic trees.
